# Functional impairment in patients with myotonic dystrophy type 1 can be assessed by an ataxia rating scale (SARA)

**DOI:** 10.1007/s00415-017-8399-x

**Published:** 2017-02-06

**Authors:** Giovanni DiPaolo, Cecilia Jimenez-Moreno, Nikoletta Nikolenko, Antonio Atalaia, Darren G. Monckton, Michela Guglieri, Hanns Lochmüller

**Affiliations:** 1 Keel University School of Medicine, Stoke-on-Trent, UK; 2 John Walton Muscular Dystrophy Research Centre, Newcastle University, Newcastle upon Tyne, UK; 3 Institute of Molecular, Cell and Systems Biology, College of Medical, Veterinary and Life Sciences, University of Glasgow, Glasgow, UK; 4 Newcastle Upon Tyne Hospitals NHS Foundation Trust, Newcastle upon Tyne, UK

**Keywords:** Myotonic dystrophy, Falls, DM1, Balance

## Abstract

Myotonic dystrophy type 1 (DM1) is not characterised by ataxia per se; however, DM1 and ataxia patients show similar disturbances in movement coordination often experiencing walking and balance difficulties, although caused by different underlying pathologies. This study aims to investigate the use of a scale previously described for the assessment and rating of ataxia (SARA) with the hypothesis that it could have utility in DM1 patients as a measure of disease severity and risk of falling. Data from 54 DM1 patients were pulled from the PHENO-DM1 natural history study for analysis. Mean SARA score in the DM1 population was 5.45 relative to the maximum score of eight. A flooring effect (score 0) was observed in mild cases within the sample. Inter-rater and test–retest reliability was high with intraclass coefficients (ICC) of 0.983 and 1.00, respectively. Internal consistency was acceptable as indicated by a Cronbach’s alpha of 0.761. Component analysis revealed two principle components. SARA correlated with: (1) all measures of muscle function tested, including quantitative muscle testing of ankle dorsiflexion (*r* = −0.584*), the 6 min walk test (*r* = −0.739*), 10 m walk test (*r* = 0.741*), and the nine hole peg test (*r* = 0.602*) and (2) measures of disease severity/burden, such as MIRS (*r* = 0.718*), MDHI (*r* = 0.483*), and DM1-Activ (*r* = −0.749*) (*p < 0.001). The SARA score was predicted by an interaction between modal CTG repeat length and age at sampling (*r* = 0.678, *p* = 0.003). A score of eight or above predicted the use of a walking aid with a sensitivity of 100% and a specificity of 85.7%. We suggest that further research is warranted to ascertain whether SARA or components of SARA are useful outcome measures for clinical trials in DM1. As a tool, it can be used for gathering information about disease severity/burden and helping to identify patients in need of a walking aid, and can potentially be applied in both research and healthcare settings.

## Introduction and objective

Myotonic dystrophy type 1 (DM1) is the most common inherited neuromuscular disorder in adults affecting approximately one in 8000 Caucasians worldwide [1]. DM1 is caused by an unstable expanded CTG repeat in the *DMPK* gene that leads to a multi-systemic and clinically heterogeneous disorder. Some of the most frequent clinical features include: myotonia, progressive muscle weakness, fatigue and daytime sleepiness, cardiac abnormalities, and psycho-cognitive disturbances [1–4].

The size of the CTG expansion mutation impacts directly on disease severity, progression, and age of onset. The most common phenotype classification includes four categories: mild (or late onset, >40 years); classic (adult, 11–40 years); childhood (early onset, 1–10 years); and congenital (severe, <1 year). However, there is no exact threshold between these groups’ phenotype and genotype [5–9].

A healthy neuromuscular system functions in a coordinated manner to achieve a deliberate and smooth voluntary movement; ataxia is a known lack of this controlled coordination. The classic adult-onset ataxias share several features with DM1, including slowly progressive gait disturbance [10–13], dysarthria [2], abnormal motor control, and reduced balance [12–14]. However, the classic ataxias result as a consequence of cerebellar dysfunction, or a damaged nervous system, whereas in DM1, these symptoms are most obviously caused by muscle weakness [14, 15]. Besides the impact on daily life activities, a major functional consequence and complication of these shared ataxia-like features is falls [14–17].

A systematic assessment of ataxia-like symptoms in patients with DM1, or in any other neuromuscular disease, has not been previously assessed. However, assessing these altered movement patterns in DM1 could give an indication of the level of impairment and how this might be interfering with the patient’s daily life, and possibly lead to a novel measure of risk of falls as it has been for other diseases [18].

Other effective motor-performance assessment tools have been proposed as outcome measures for DM1 [19, 20]. However, the practicability of these tests in clinical practice is limited by the availability of evaluators experienced with DM1, familiarity with the tests, time, and clinic facilities. This study aims to test the scale for the assessment and rating of ataxia (SARA), created by Schmitz et al. [21, 22], with the overall aim of identifying and validating SARA in measuring aspects of disease severity in DM1 as a simple, and time-saving tool SARA has already been identified as a reliable index of gait status and daily live independence in ataxic stroke patients [18].

## Methods

### Sample

This study assessed data from 54 patients who had been recruited into the ongoing PHENO-DM1 study (Myotonic Dystrophy Type 1 Deep Phenotyping to Improve Delivery of Personalized Medicine and Assist in the Planning, Design and Recruitment of Clinical Trials) [23], a multicentre observational cohort study that aims to assess the natural disease progression of DM1 and identify outcome measures that efficiently represent the disease phenotype. This cohort represents the first 54 patients recruited to one of the sites (Royal Victoria Infirmary—Newcastle Upon Tyne Hospitals NHS Foundation Trust). The inclusion criteria for this study were: (1) 18 years or older; (2) genetic confirmation of DM1; and (3) ability to consent and participate throughout the entire study, including walking tests (able to complete the 10 m walk test with no other person’s assistance as a minimum requirement). Patients were classified as mild if they met two of the three following criteria: (1) age of onset of 40 years or more; (2) modal allele length of less than 200 CTG repeats; or (3) a muscle impairment (MIRS) score of one or two [24].

### Procedures

All the following tests and outcome measures come from PHENO-DM1 study visits. The SARA test includes eight performance-based items (gait, stance, sitting, speech disturbance, finger chase, nose–finger test, fast alternating hand movements, and heel-shin slide). Each item has an independent scoring range, but applicable to all items a score of zero implies no dysfunction, and an increasing score represents a more severe degree of ataxia, acquiring the maximum score if an individual is unable to complete the item task at all. The scores for all items are summed, and a score out of a maximum score of 40 is given. There is no need of training or additional equipment for this assessment [21]. However, for this study, assessors were instructed to follow closely each item scoring system regardless of the aetiology of the impairment.

Upon enrolment, all patients performed the SARA test and were scored by one of three assessors, all of which had previous experience with DM1 patients.

For assessment of reliability, 14 of the 54 patients were rated twice by the same assessor at the beginning and once at the end of the study visit. These 14 patients were also independently assessed by a second assessor during the day. The assessors were blinded as to each other’s results.

The main outcomes considered for comparisons were: (1) muscle capacity (including quantitative muscle testing (QMT) of hand-grip strength, knee extensors; hip flexors and ankle dorsiflexors, plus the Muscular Impairment Rating Scale (MIRS) [24]); (2) functional performance, including: the nine hole peg test (9HPT); 6 min walk test (6MWT); 30 s sit and stand (TSST); 10 m walk test (10 MWT); and the 10 m walk/run test (10MW/RT); and (3) patient reported outcome measures (PROM) which included the DM1-Activ^a^ Rasch-built scale [25], which assesses a patient’s performance in daily life activities and the Myotonic Dystrophy Health Index (MDHI)^b^ [26], which scores the impact of a wide variety of disease related signs and symptoms on a patient’s life.

Additional variables considered relevant for this analysis were: (1) the estimated progenitor allele length (CTG repeats) [27, 28], which were available for 26 (50%) patients who have also been part of the OPTIMISTIC cohort [29]^c^; (2) the use of a walking aid(s) when performing the functional tests; (3) patient’s reported experienced falls over the last week, month, and year and associated injuries (e.g., head injury, fracture, etc) for 24 patients; and (4) age at sampling.

This research is covered under the ethical approval of the PHENO-DM1 study approved by The Newcastle and North Tyneside Ethics committee (Reference: NE/15/0178)^d^.

### Statistical analysis

Normal distribution was explored utilising the Shapiro–Wilk test based on the size of this sample. Inter-rater and test–retest reliability was expressed using intraclass correlation coefficients (ICC) based on a two-way mixed-effect model. Internal consistency was deemed acceptable with a Cronbach’ alpha coefficient of 0.7 or higher [22]. A principal component analysis was performed using varimax rotation to extract components with eigenvalues above one. Associations between SARA scores and other variables were tested. Pearson’s and Spearman’s Rho correlation tests were applied for normally and not normally distributed variables, respectively. An additional linear regression test was performed in search of the square *R* values to identify any causal relationship between variables. Construct validity was accepted when values fell between the range of 0.4–0.8.

Receiver operating characteristic (ROC) curves were constructed and based on the highest combination of sensitivity and specificity to determine a cut-off point (if any) that could differentiate the patient’s walking status (with or without a walking assistive device). For all tests, *p* values of <0.05 were considered statistically significant.

## Results

### Sample demographics

From the total cohort, three patients were excluded: two as they were unable to complete the SARA due to physical constraints (these patients were unable to lie flat during the assessment of the heel-shin slide) and another one due to comorbidity with Charcot–Marie Tooth disease. Leaving a final sample of 51 patients (30 male) formed: of 10 mild cases; 35 classic; and six considered early (childhood) onset. Nine patients were using orthotics at visit and ten, assistive devices (one side stick/cane) (Table 1).

**Table 1:** Sample demographics

	Mean (SD)	Range (min to max)
Age (years)	47.7 (12.6)	18–77
Body mass index (kg/m^2^)	25.7 (6.8)	16.2–41.7
Time since disease onset (years)	19.5 (11.8)	5–53
MIRS (1–5)	3 (1.2) 1 = 12% 2 = 31% 3 = 18% 4 = 31% 5 = 8%	1–5
Modal CTG repeat length in blood	564.5^a^ (324.9)	80–1130

^a^Only from those available

### SARA scoring

The mean SARA for this DM1 sample was 5.5 SD ± 4.5 (range 0–18.5). No patient scored a maximum score on any of the scale items. Items sitting, finger chase, and nose finger test ever scored between zero and two out of a possible four, with ten patients scoring <1. There was a statically significant difference in the mean (SD) SARA scores between genders [female 3.8 (+3.1) vs. male 6.6 (±5) *p* < 0.05] and phenotype groups [mild 2.5 (±2.2) vs. classic 6.2 (±4.6) *p* < 0.005].

### Reliability

A two-way mixed-effect model determined the single measures of the inter-rater and retest reliability with ICCs of 0.916 and 1.0, respectively.

### Validity

Cronbach alpha was acceptable at 0.761 for SARA score. This was indicated to improve to 0.765 or 0.786 if the finger chase or nose–finger test items were deleted, respectively. Principal component analysis revealed two components with an eigenvalue above one. The first component had an eigenvalue of 3.36 and was responsible for 42% of the variance present; it was also shown to affect gait, stance, sitting, speech, and heel-shin slide (items which represent more ataxia affecting balance and lower limb function predominantly). The second component had an eigenvalue of 1.61, responsible for 20.2% of total variance and shown to affect items sitting, finger chase, nose finger test, and heel-shin slide (items related to ataxia affecting upper limb function) (Table 2).

**Table 2:** Proportions of variance for each component within the items of SARA

Item	Component revealed	
	1	2
1 Gait	0.900	
2 Stance	0.812	
4 Speech	0.800	
7 Fast alternating hand movement	0.632	
8 Heel-shin slide	0.585	0.425
5 Finger chase		0.807
6 Nose–finger test		0.734
3 Sitting	0.487	0.631

### Convergent validity

#### Muscle capacity assessment

SARA correlated significantly with all muscle strength results, with the MIRS and the QMT ankle dorsiflexion as the strongest (*ρ* of ≥0.5 at a significance of *p* < 0.001). The functional assessments (6MWT, 10MWT, 10MRT, TSST, and 9HPT) also showed a strong and statistically significant correlation score (Table 3). Assessments of ambulation resulted in the highest square *R* values for the SARA score.

**Table 3:** Correlations of SARA scores with muscle function tests: quantitative muscle test (QMT), 6 min walk test (6MWT), 30-s sit and stand test (TSST), nine hole peg test (9HPT), 10 m walk test (10MWT), and the 10 m run test (10MRT)

Measure	Mean (SD)	Correlation	Linear regression	Sig (*p* value)
		*R*	Adjusted square *R*	
Hand-grip strength (kg)	14 (9.3)	−0.505	0.239	<0.001
Wrist extension (lbs)	14.4 (9.7)	−0.475	0.208	0.001
Ankle dorsiflexion (lbs)	19.3 (15.8)	−0.584	0.324	<0.001
Knee extension (lbs)	47.8 (20.8)	−0.308	0.085	0.023
Hip flexion (lbs)	29.0 (16.8)	−0.184	0.004	0.367
6MWT (m)	442.5 (177.8)	−0.739	0.472	<0.001
TSST (repetitions)	11.3 (6.1)	−0.509	0.216	0.001
9HPT (s)	22.5 (10.6)	0.602	0.348	<0.000
10MWT (s)	8.6 (2.9)	0.741	0.539	<0.001
10MRT (s)	4.6 (3.3)	0.668	0.434	<0.001
MIRS (stages 1–5)	3.0 (1.2)	0.718	0.506	<0.001

#### Patient reported outcome measures (PROM)

The analysis showed a moderate-to-strong correlation for both patient reported outcomes. The MDHI total score produced a correlate of 0.48 and the DM1-Activ of −0.75, both at a significance of *p* < 0.0001 (Table 4).

**Table 4:** Correlations of SARA with the patient reported outcomes: DM1-Activ Rasch-built scale and Myotonic Dystrophy Health Index (MDHI)

PROM	Mean (SD)	Correlation	Linear regression	Sig (*p* value)
		*R*	Adjusted square *R*	
MDHI—total	26.5 (21.1)	0.483	0.218	<0.001
MDHI—cognition	18.9 (19.4)	0.095	−0.012	0.51
MDHI—vision	15.6 (22.7)	0.056	−0.018	0.7
MDHI—myotonia	31.7 (29.0)	0.595	0.34	<0.001
MDHI—fatigue	42.2 (34.7)	0.435	0.172	0.002
MDHI—mobility	15.6 (30.3)	0.627	0.380	<0.001
DM1-Activ	2.74 (2.7)	−0.749	0.552	<0.001

### Age and CTG repeat length

Surprisingly, there was no detectable correlation between SARA and age at sampling (*r* = 0.131, *p* = 0.525). Likewise, there was only a marginally significant correlation between SARA and modal CTG repeat length at sampling (*r* = 0.377, *p* = 0.057). This most likely reflects anticipation mediated sampling bias with a highly significant inverse correlation between age at sampling and modal CTG repeat length (*r* = −0.528, *p* = 0.006). Indeed, there was a highly significant interaction between age at sampling and modal CTG repeat length (*r* = 0.678, *p* = 0.003) accounting for ~39% of the variation in SARA score (adjusted *r* ^2^ = 0.389).

### Falls

Falls history of 24 patients was retrospectively collected, and for this cohort, their SARA score correlated with number of falls experienced in the previous month (*r* = 0.436, *p* < 0.05), but did not correlate with the number of falls that had occurred in the last week or with number of injuries as a result of falls.

### Use of a walking aid

**Fig. 1:**
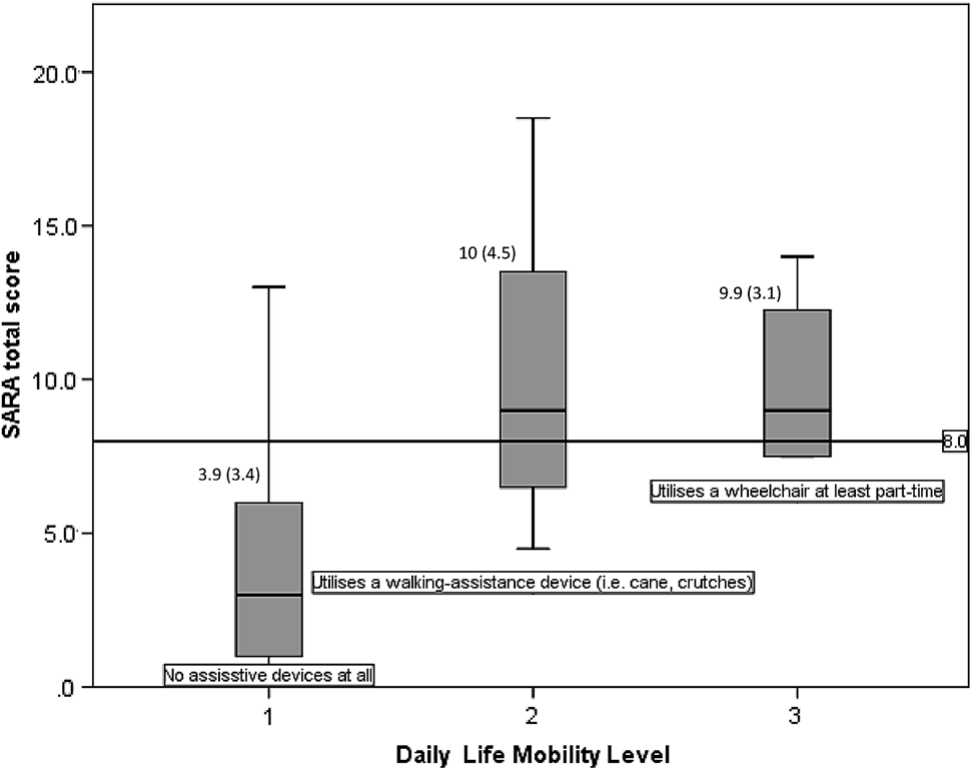
SARA score according to gait status in daily life activities. Values are mean (SD)

An ROC curve was generated for patients with and without a walking aid during their walking tests based on SARA score. The area under the curve was 0.962. An optimal combination of sensitivity and specificity (100 and 85.7%, respectively) gave a threshold SARA score of 8 or above for the identification of DM1 patients in use of a walking aid. With regard to the reported gait status in daily life activities, the mean SARA scores for (1) totally independent, (2) cane/crutch/walker dependent gait, and (3) wheelchair (at least part-time dependent) ambulation were 3.9, 10, and 9.9, respectively, with a significant difference between the independent gait mean scores against the dependent (Fig. 1).

## Discussion

SARA was originally intended and validated as an assessment measure for patients with spinocerebellar ataxia. Notably, average CTG repeat lengths in most postmortem brain regions of DM1 patients are typically thousands of repeats longer than the inherited allele length and this very likely contributes to the wide range of neurological symptoms observed in this disorder [30–32]. However, such large expansions are not observed in the cerebellum, where the CTG repeat appears to be possibly even more stable than observed in blood [30–32]. Of course, this does not exclude a cerebellar dysfunction in DM1, but it is not a frequent feature of myotonic dystrophy. This does not, however, mean that ataxia-like signs are not present via the means of a different pathological process (e.g., muscle weakness, impaired balance, or intermittent myotonia) and that these cannot be measured and quantified. Peripheral neuropathies could also be a possible cause for alteration in SARA scoring; to assess this formally nerve conduction studies would be required, this is beyond the scope of this study. Furthermore, no participants had brain MRI performed to look for evidence of cerebral lesions. The present study aimed to investigate the use of an ataxia scale, SARA, to measure these features and the association of this score to disease burden.

The sample size used in this study was comparable to previous validation studies of SARA in patients with ataxia [18, 22]. The overall SARA scores for patients in this sample were lower than those in the previous studies using SARA. This can be attributed to the combination of the sample containing ten mild cases, which tend to be unaffected in terms of muscular impairment as displayed by a ceiling effect within our results, and the exclusion of non-ambulant DM1 patients who would have represented a more severely affected group of patients. The generalizability of these results, therefore, may be limited to adult patients still able to walk independently for at least ten meters. However, this cohort represents a DM1 patient subpopulation likely to be recruited into clinical trials.

The results of this study indicate good clinicometric properties for the use of SARA in patients with DM1. Both retest reliability and inter-rater reliability were very high with similar scores to previous publications in other indications [18, 22]. However, the perfect ICC value of 1.00 for retest reliability may be attributed in part to the fact that the repeat testing occurred on the same day with assessors remembering their scores from earlier in the day. However, after achieving a high inter-rater reliability in this study and the proven reliability of SARA in other ataxia patients in previous studies, we may consider SARA to be a reliable and reproducible scale across different disease entities, not limited to classical ataxias [22].

A good internal consistency was displayed, with a Cronbach alpha score of 0.761. The change in Cronbach alpha after removing either of the finger chase or nose finger test items was negligible, and their inclusion in this particular test may be valuable for two reasons. The first being that the degree to which ataxia affects the upper limbs will affect the degree of difficulty to which a patient will find performing tasks, such as getting dressed, washing themselves or cooking, etc., which ultimately will have an effect on their quality of life. Second, effective coordination of upper limb movement provides a protective mechanism for patients when they fall (for example, patients who outstretch an arm when [33] falling to the ground may prevent the occurrence of significant injuries). Further investigation on this topic is being considered and Rasch analysis may improve the internal consistency of the scale for this particular population. However, any change or adaptation of the scale would have an impact on the comparability of the new datasets with historical control and disease populations [34].

The component analysis identified two principal components with an eigenvalue above one. It is difficult to ascertain precisely what these components truly reflect, and our speculation is based on the following observation. The most prominent principal responsible for 42% of the variance seems to be derived from items affecting lower limbs and general balance (i.e., gait, stance, sitting, and heel-shin slide) as opposed to the second component which is solely responsible for variance in the finger chase and nose–finger tests which derives from upper limb function. It is unlikely that either component is cerebellar ataxia as this would likely be a component causing variance in all eight items as opposed to a select number, but again, confirmatory testing, such as neurophysiology or brain MRIs, would be required to support this conclusion. We would suggest that both these differing components are likely to be differing distributions of muscle weakness, because myotonic dystrophy is a heterogeneous disease and may affect patients differently including the distribution of muscle weakness [13, 15].

Ataxia is a clinical feature that has not been identified in any form previously in patients with DM; however, the authors predicted that the aetiology of these features of impaired function and signs of imbalance in this condition are at least in part a result of muscular weakness. Patient’s with a higher SARA score performed significantly worse on all tested measures of muscle function, including muscle strength, balance, gait speed, and stamina. A significant finding was the strong correlation of the SARA score with ankle dorsiflexion strength as this muscle group that has previously been linked significantly to falls risk in DM1 patients [14–17]. We did not attempt to cross-validate SARA against other balance scores, such as the Berg balance score, the mini-BESTest, or the Step test for dynamic balance [12, 14, 19, 20]. This would be recommended before suggesting the application of SARA as a balance score more broadly.

The impact that these motor signs can have in daily life activities and disease burden was assessed with a correlation test against DM1-Activ and MDHI reported outcomes and both questionnaires showed a strong overall association with the SARA total score. For the MDHI, the strongest *r* values were for the sets related to mobility, when, compared to the lack of correlation with the subscales of vision and cognition, it gives signs of good discriminant validity. The correlation of SARA to dependence level of daily life activities has been shown before for ataxic stroke patients [18].

As expected, SARA scores were highly positively correlated with age and CTG modal allele length. However, these effects were only revealed in testing for an interaction between these two factors. These data further highlight the confounding effects of anticipation mediated age at sampling ascertainment bias that results in sampling mildly affected parents at a much older age relative to their more severely affected offspring.

The analysis of predicted falls may be limited as this was based on a retrospective interview of the most recent week and month fall rate and not applicable to the whole sample, which may be liable to recall bias. Moreover, answers may have been influenced by the interviewer who was not blinded to the SARA score. It is necessary to follow this finding and search for confirmation with a prospective longitudinal data recollection. However, several variables showed strong correlation with SARA that has previously been identified as fall predictors in DM1: ankle dorsiflexion, knee extension and hip strength, and the time to walk 10 m at a comfortable fast speed [14, 15, 17]. A prospective recollection of falls incidence previous to their follow-up visit might corroborate the validity of these results.

SARA is a clinical scale that corresponds well to patient’s gait status and activities of daily living score [18] assessing symptoms, such as gait performance, balance, and movement coordination. In DM1, it could help to identify patients with higher risk of falling and the requirement of supplementary walking tools [18, 33]. We used the ROC curve for patients with or without walking aid against SARA score; a score of eight or above was used to predict which patients were using an assistive device. This score was selected as it achieved the highest combination of sensitivity and specificity possible (sensitivity 100% and specificity 85.7%). There were six patients with SARA scores of eight or more with no records of an assistive walking device during their visits. However, after reviewing this cohort of six patients with the physiotherapy team, we discovered that: two (33%) had experienced a fracture as a result of a fall in the recent past and three (50%) had been previously advised to use a walking aid (for example, to use when walking longer distances). Kim et al. [18] previously defined the following cut-off values to identify the gait status on their ataxic cohort: scores of 8 or lower for independent gait, 11.5 or lower for Q-cane gait, and 12.25 or lower for walker assisted gait. Because of the ambulatory conditions of this study’s cohort, the conclusions for this topic have been limited to a cutoff that identifies independent gait status from gait with assistive device. Still, these results may highlight how SARA can be considered by clinicians as a risk predictor, with a score of eight or above as a guideline to implement a walking aid as a prophylactic intervention, hopefully preventing falls and aiding in activities of daily living.

## Summary

The present study aimed to investigate an ataxia scale in patients with DM1, assessing its validity and reliability as a possible tool for clinicians working with DM1 patients. SARA could be a practical tool to assess disease severity, falls risk, and the requirement of a walking aid in DM1.

SARA has met the criteria necessary to deem it reliable and valid with high retest and inter-rater reliability, good convergent validity, and acceptable internal consistency. SARA has been shown to correlate with multiple gold standard health outcome measures specific to patients with DM1 (such DM1-Activ, MDHI) and markers of disease severity. Furthermore, this study has revealed a SARA score of eight or above that can predict the use of a walking assistive device.

Future prospective research in a larger sample, including other validated tests and assessments for falls, followed by RASCH analysis, is warranted to further explore the validity and utility of SARA or components of SARA in DM1. Follow-up of these patients within the PHENO-DM1 study at regular intervals will also help to determine the sensitivity of SARA to detect disease progression. It is unclear whether the scale can be used in a wider range of neuromuscular diseases, children, or older adults.
